# Replication of somatic micronuclei in bovine enucleated oocytes

**DOI:** 10.1186/1747-1028-7-23

**Published:** 2012-11-22

**Authors:** Natalia Canel, Romina Bevacqua, María Inés Hiriart, Daniel Salamone

**Affiliations:** 1 Laboratorio Biotecnología Animal, Departamento de Producción Animal, Facultad Agronomía, Universidad de Buenos Aires, Av. San Martín 4453, C1417DSE, Buenos Aires, Argentina

**Keywords:** Micronuclei, Oocyte, Chromosomes, Transgene

## Abstract

**Background:**

Microcell-mediated chromosome transfer (MMCT) was developed to introduce a low number of chromosomes into a host cell. We have designed a novel technique combining part of MMCT with somatic cell nuclear transfer, which consists of injecting a somatic micronucleus into an enucleated oocyte, and inducing its cellular machinery to replicate such micronucleus. It would allow the isolation and manipulation of a single or a low number of somatic chromosomes.

**Methods:**

Micronuclei from adult bovine fibroblasts were produced by incubation in 0.05 μg/ml demecolcine for 46 h followed by 2 mg/ml mitomycin for 2 h. Cells were finally treated with 10 μg/ml cytochalasin B for 1 h. *In vitro* matured bovine oocytes were mechanically enucleated and intracytoplasmatically injected with one somatic micronucleus, which had been previously exposed [Micronucleus- injected (+)] or not [Micronucleus- injected (−)] to a transgene (50 ng/μl pCX-EGFP) during 5 min. Enucleated oocytes [Enucleated (+)] and parthenogenetic [Parthenogenetic (+)] controls were injected into the cytoplasm with less than 10 pl of PVP containing 50 ng/μl pCX-EGFP. A non-injected parthenogenetic control [Parthenogenetic (−)] was also included. Two hours after injection, oocytes and reconstituted embryos were activated by incubation in 5 μM ionomycin for 4 min + 1.9 mM 6-DMAP for 3 h. Cleavage stage and *egfp* expression were evaluated. DNA replication was confirmed by DAPI staining. On day 2, Micronucleus- injected (−), Parthenogenetic (−) and *in vitro* fertilized (IVF) embryos were karyotyped. Differences among treatments were determined by Fisher′s exact test (p≤0.05).

**Results:**

All the experimental groups underwent the first cell divisions. Interestingly, a low number of Micronucleus-injected embryos showed *egfp* expression. DAPI staining confirmed replication of micronuclei in most of the evaluated embryos. Karyotype analysis revealed that all Micronucleus-injected embryos had fewer than 15 chromosomes per blastomere (from 1 to 13), while none of the IVF and Parthenogenetic controls showed less than 30 chromosomes per spread.

**Conclusions:**

We have developed a new method to replicate somatic micronuclei, by using the replication machinery of the oocyte. This could be a useful tool for making chromosome transfer, which could be previously targeted for transgenesis.

## Background

Manipulation of genetic information has been under investigation for many years. However, manipulation of chromosomes has only been attempted by the generation and use of artificial chromosomes capable of carrying large DNA sequences, e.g. YAC’s (yeast artificial chromosomes) [1,2] and HAC’s (human artificial chromosomes [3,4]. Although such molecules can carry large fragments of DNA, the isolation and manipulation of individual entire chromosomes has never been achieved.

In 1974, Ege and Ringertz developed a methodology for generating cells with small amounts of DNA [5]. These cells, having only one or a few chromosomes (microcells) were first fused with mitotic cells to generate hybrid karyotypes by Schor et al. [6]. In 1977, Fournier and Ruddle [7] designed the microcell-mediated chromosome transfer technique (MMCT) which is still used to introduce a low number of chromosomes into host somatic cells [8,9]. This technique has been used in several ways, mainly for the mapping of genes, like tumor suppressor genes [10-15], telomerase suppressor genes [16-19], senescence inducing genes [20-22], and genes involved in DNA repair pathways [23-25]. It has also been used to study the effect of genomic imbalances on chromosome- specific gene expression patterns and the behavior of polysomies in different cell lines [26,27], and for analysis of genomic imprinting [28-30].

Recipient cell types used for MMCT can be somatic cells, embryonic carcinoma (EC) or embryonic stem (ES) cells, which are employed to study *in vitro* or *in vivo* effects of the transferred chromosomes [31]. This method consists of the chemical micronucleation of a donor cell line whose chromosome under study contains a dominant selectable marker. Treated cells develop several micronuclei, which contain one or, at most, a few chromosomes. Microcells are then isolated by high speed centrifugation in the presence of cytochalasin B. After a filtration process, microcells with the smallest number of chromosomes are collected and finally fused with recipient somatic cells. The dominant selectable marker previously introduced into donor cells allows recognition of resulting hybrid cell lines which have incorporated the chromosome of interest [32,33]. In this way, MMCT can be considered as a simple way to manipulate an entire chromosome as a structural unit. Nevertheless, it is not possible to select and replicate such chromosomes individually.

It is well known that the oocyte has the capacity to replicate varied numbers of chromosomes. It has been demonstrated that haploid, polyploid and mixoploid embryos can cleave and also reach the blastocyst stage in the bovine [34,35], porcine [36] and human [37-40]. Polyploid blastocysts have also been produced in rabbits [41] and mice [42]. Additionally, it has been established that the mammalian oocyte can replicate not only the genetic information from the gametes [43,44], but also from somatic cells [45,46], including cells from different species [47]. Mouse first polar bodies have also been used as nuclear donors [48]. After injection of polar bodies, enucleated oocytes were subjected to ICSI and fertile offspring were obtained following embryo transfer to foster mothers [48]. In addition, bovine embryos reconstituted with non enucleated oocytes showed similar cleavage and blastocyst rates to those reconstituted with enucleated oocytes [49]. On the basis of these previous results, we can propose that the oocyte is capable of replicating a wide range of numbers and types of chromosomes.

The MMCT technique is still widely used, even though the methodology has not undergone any major technical changes since it was developed. In the present work, we gave a new focus to this technique by using the bovine ooplast to copy a single or low number of chromosomes. With the aim of generating several copies of individual chromosomes to be able to manipulate them, we combined part of the MMCT technique with somatic cell nuclear transfer (SCNT). The new methodology that we designed consists of injecting one somatic micronucleus into an enucleated oocyte and inducing the injected oocyte to cleave. Each blastomere from the injected oocytes should have a copy of the donor micronucleus, giving rise to the replication of an individual chromosome or of a low number of chromosomes.

We also tested the ability of somatic micronuclei to express a transgene after being injected into an ooplast. Recently, we demonstrated a spontaneous and quick interaction between DNA and donor cells or fragments of ooplasm surrounded by oolema (vesicles) [50]. In this study, cumulus cells previously exposed to pCX-EGFP plasmid for 5 min were injected into enucleated bovine oocytes and gave rise to *egfp* expressing embryos by SCNT. Vesicles exposed to pCX-EGFP were injected into MII oocytes and IVF embryos, resulting in the production of parthenogenetic and IVF *egfp* expressing embryos respectively. On the basis of these results, we hypothesize that the membranes surrounding micronuclei are also capable of binding DNA, resulting in the production of *egfp* expressing embryos.

In summary, after chemical activation, cleavage occurred, and some micronuclei were replicated. A low number of chromosomes (1–13) was detected. Additionally, a small proportion of micronuclei- injected oocytes showed transgene expression. The possibility of generating embryos containing only one chromosome would allow us to genetically modify, identify and transfer individual chromosomes, while multiplying them into many blastomeres. In this way, it would be possible to confirm which chromosome has been replicated by the oocyte through biopsy and molecular analysis of only one of the blastomeres. This method constitutes a first approach to individual chromosome manipulation.

## Results and discussion

### Induction of somatic micronuclei

With the aim of inducing the formation of somatic micronuclei, several chemical treatments were tested on primary cell cultures. Although there are no previous reports of the generation of micronuclei in bovine cells, we assayed different treatments to induce a block in metaphase, on the basis of human or mouse protocols [7,10,26,51]. Somatic cells were treated with 0.05 or 0.1 μg/ml demecolcine (DMC); or 0.05 μg/ml colchicine (Col) for 46 or 48 h, followed or not by incubation with 2 mg/ml mitomycin (Mit) for 2 h. The six treatments assayed were named DMC 0.05 μg/ml; DMC 0.05 μg/ml + Mit; DMC 0.1 μg/ml; DMC 0.1 μg/ml + Mit; Col 0.05 μg/ml; and Col 0.05 μg/ml + Mit. A total of 300 to 500 cells per treatment were evaluated. No differences were found between groups in percentages of cells that became micronucleated over total number of cells (Table 1), except for DMC 0.05 μg/ml + Mit, which showed a significantly higher value (21.75 vs. 3.25 to 10%) (p≤0.05). All treatments induced similar numbers of micronuclei per cell, varying from 2 to 20. Rates of micronucleation were very low compared to mouse lines from previous studies. This might be due to the cell type used as donor, because not all cells are capable of micronucleating with the same efficiency (Eric Shoubridge, personal communication). In mice, the cell line mostly used for MMCT experiments is the A9 line, which has low tumorigenicity and lacks hypoxanthine phosphoribosyltransferase and adenine phosphoribosyltransferase [52]. It has been s reported to have rates of micronucleation of 80-90% after a 48 h treatment with colcemide [7]. Because of its high efficiency, it has been widely employed for generating microcells [8,26,53]. On the basis of the low rates of micronucleation obtained, we decided to treat cells with mitomycin, to complete mitotic arrest, as described by De Lonlay et al. [54] who synchronized transformed rodent cell lines. DMC 0.05 μg/ml + Mit treatment produced the best results, and was therefore chosen to generate micronucleate cells for further experiments.

**Table 1:** Evaluation of different combinations of spindle inhibitors and mitomycin for the induction of somatic micronuclei

**Spindle inhibitor**	**Mit**	**% Micronucleate cells / total cells**	**Media of micronuclei / cell**
**DMC 0.05 μg/ml**	-	10^a^	7
	+	25.5^b^	7
**DMC 0.1 μg/ml**	-	6.25^a^	7
	+	4.25^a^	8
**Col 0.05 μg/ml**	-	3.75^a^	6
	+	3.25^a^	6

Ovarian bovine fibroblasts were blocked in metaphase by incubation with 0.05 or 0.1 μg/ml DMC (Demecolcine); or 0.05 μg/ml Col (Colchicine) for 46 or 48 h. In order to complete mitotic arrest, some cells were then incubated with 2 mg/ml Mit (Mitomycin) for 2 h.

^a,b^: Values with different superscripts in the same column differ significantly (p≤0.05).

### Cleavage and *egfp* expression of enucleated and micronucleus injected bovine oocytes

On the basis of experiment 1, DMC 0.05 μg/ml + Mit treatment was employed to generate micronucleate cells. Micronuclei previously incubated or not with pCX-EGFP were injected into enucleated oocytes. Cleavage rates were evaluated (Table 2). Micronucleus- injected (+), Parthenogenetic (+) and Parthenogenetic (−) groups showed higher cleavage rates than the Micronucleus- injected (−) treatment [93/108 (86.11%), 111/136 (81.62%) and 160/186 (86.02%) respectively vs. 80/108 (74.07%)] (p≤0.05). Cleavage rates of the Enucleated (+) group [78/105 (74.3%)] did not differ from Micronucleus- injected (−), and Parthenogenetic (+) treatments (p≤0.05). Cleavage rates were similar between groups (Table 2), except for the Micronucleus- injected (−) group, which showed a lower rate (81.62 to 86.11 vs. 64.91%) (p≤0.05). It is important to notice that rates of cleavage are difficult to discern from those of fragmentation for the Micronucleus- injected and Enucleated groups. We considered as cleaved all embryos with more than 2 blastomeres, independent of their symmetry. This was based on the fact that embryos reconstituted with micronuclei showed both symmetric and asymmetric cells.

**Table 2:** **Cleavage and*egfp*expression rates of Micronucleus- injected bovine oocytes**

**Treatment**	**pCX-EGFP**	**N**	**Cleavage (%)**	***egfp*expressing embryos (%)**
**Micronucleus- injected**	-	108	80 (74.07)^a^	-
	+	108	93 (86.11)^bc^	2 (2.15)^a^
**Parthenogenetic**	-	186	160 (86.02)^bc^	-
	+	136	111 (81.62)^ac^	43 (38.74)^b^
**Enucleated**	+	105	78 (74.29)^a^	0^a^

Micronucleus- injected (−) (embryos generated from enucleated oocytes injected with one micronucleus and parthenogenetically activated), Micronucleus- injected (+) (embryos generated from enucleated oocytes injected with one micronucleus previously incubated with pCX-EGFP and parthenogenetically activated), Parthenogenetic (−) (parthenogenetically activated oocytes), Parthenogenetic (+) (parthenogenetically activated oocytes injected with pCX-EGFP), Enucleated (+) (enucleated oocytes injected with pCX-EGFP and parthenogenetically activated). *egfp* expressing embryos: embryos with 2 or more *egfp* expressing blastomeres (expressing embryos/cleaved embryos).

^a,b,c^: Values with different superscripts in the same column significantly differ (p≤0.05).

In experiment 2, *egfp* expression was also analyzed. Interestingly, a low number of Micronucleus- injected (+) embryos showed *egfp* expression. Expression levels were significantly lower than those observed for the Parthenogenetic (+) control (2.15 vs. 38.74% respectively) and they did not differ from the Enucleated (+) control, which did not show *egfp* expression at all (p≤0.05). From these data, we can conclude that it is not necessary for the oocyte to have an entire nucleus to allow the transgene to be expressed. According to our hypothesis, the membranes surrounding micronuclei could have bound DNA, or the transgene could also have been transferred in the media during the injection of the micronuclei, as this is the way in which we produced the *egfp* expressing parthenogenetic control embryos. The transgene was delivered directly into the cytoplasm of the parthenotes by microinjection, before chemical activation. In a previous work done in our laboratory, eDNA alone was injected into MII oocytes in the same way and concentration of pCX-EGFP than in the present study, giving rise to a 25% of *egfp* expressing embryos at day 4 of *in vitro* development [50]. In a second study from our group, Bevacqua et al. [55] reported 50% of *egfp* expressing embryos, working under the same conditions.

Although *egfp* expression was observed in Micronucleus- injected group, rates of expression were low. This result could indicate that a non complete nucleus is not as efficient as an entire nucleus to support transgene expression. One possible explanation for the latter observation could be the unusual structure that the micronucleus adopts at the interphase stage. There are several indirect evidences that suggest that the inner nuclear membrane might have a repressor action over transcription of many genes. One of these is that heterochromatin is usually positioned near the periphery of the nucleus [56]. In our work, we used micronuclei as nuclear donors which must have a different content of chromosomes; their transcription (and also those of the transgene) could have been repressed or not, depending on the position of the chromosomes within the nuclear envelope. On the other hand, *egfp* expression observed in the Enucleated (+) group was zero, while a 38.74% rate of expression shown by the Parthenogenetic (+) control group is evidence that the presence of a nucleus is necessary for transgene expression. These results agree with Spann TP et al. [57] who reported an association between the normal structure of *Xenopus* embryo nucleus and the occurrence of transcription.

**Figure 1:**
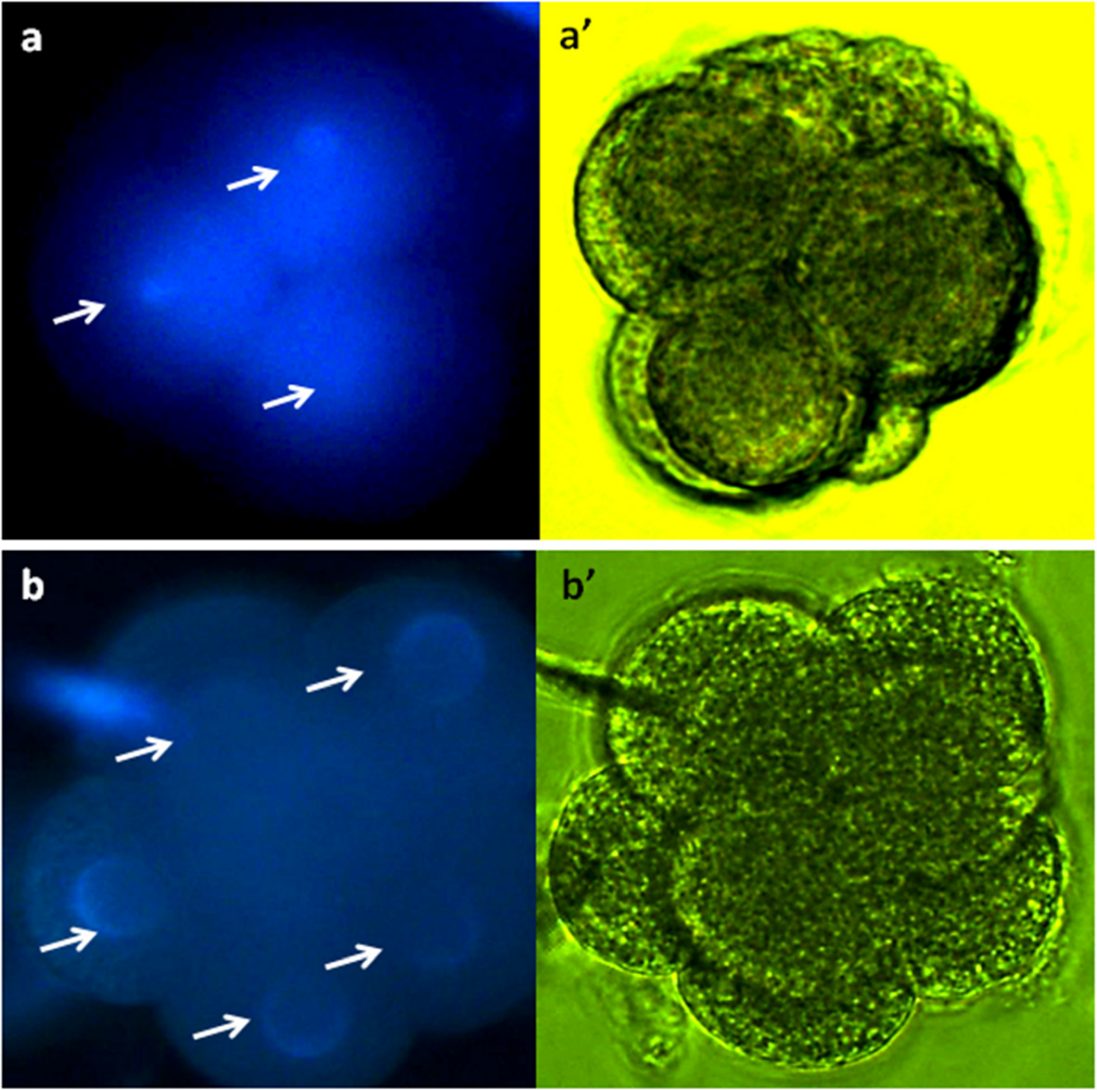
**Assessment of nuclear replication.** (**a**) Micronucleus- injected and (**b**) Parthenogenetic cleaved embryo observed under UV light after DAPI staining. (**a’** and **b’**): The same embryos under bright light. Arrows indicate DNA staining (200X).

To confirm replication of DNA, some Micronucleus- injected (−) and Parthenogenetic (−) cleaved embryos were fixed and stained with DAPI (Figure 1). The presence of more than one micronucleus per embryo was evaluated. Although rates of Micronucleus- injected (−) embryos with more than 1 nucleus (63.6%, n=22) were lower than those for the Parthenogenetic (−) group (100%, n=28) (p≤0.05), DAPI staining confirmed replication of micronuclei. From these data, we concluded that early cleavage is not dependant on the presence of a complete nucleus. These observations could be explained by the timing of zygote genome activation. In bovine embryos, low transcription activity is observed before the 4-cell stage [58-60]. However, protein synthesis is supposed to be programmed by maternal mRNA up to the 8-cell. It is between the 8-cell and 16-cell stage when the zygote genome becomes activated, and the higher transcription levels are detected [61,62]. Thus, in this context, the cytoplast could cleave without the presence of an entire nucleus. To our knowledge, this is the first report of the production of reconstituted embryos using micronuclei as nuclear donors.

### Chromosomal analysis of micronucleus injected embryos

**Figure 2:**
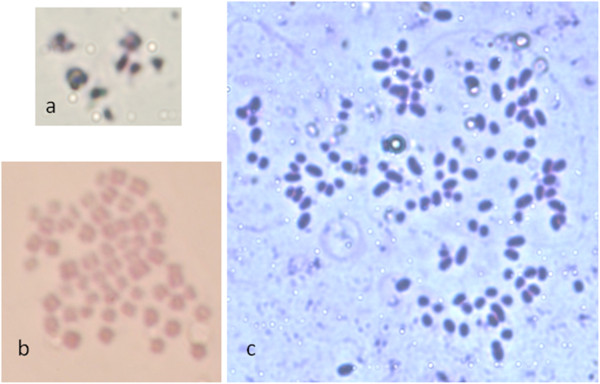
**Chromosomal analysis.** Spreads from (**a**) Micronucleus- injected (less than 1n), (**b**) IVF (2n) and (**c**) Parthenogenetic cleaved embryo (4n) treated with colchicine and stained with Giemsa (1000X).

Karyotypes of Micronucleus- injected (−) embryos were studied (Table 3 and Figure 2). Parthenogenetic (−) and IVF controls were also included. All Micronucleus- injected (−) embryos analyzed had fewer than 15 chromosomes, while none of the controls showed such results. Eighteen blastomeres from 11 Micronucleus- injected (−) embryos were counted. All evaluated embryos had at least two nuclei, at metaphase or interphase. Numbers of chromosomes per blastomere varied from 1 to 13, with a median of 7. The number of chromosomes in blastomeres from each of the eleven analyzed embryos were: 8, 8, 7, 11 and 11; 7 and 8; 10 + interphase in 4 embryos; 13 + interphase; 6 + interphase; 1 + interphase; 2 and 2; 3 and 1. Despite the efficiency of individual chromosome embryo production should be improved, these results indicate that the oocyte is capable of replicating a low number of chromosomes. It is important to note that the number of chromosomes injected into the enucleated oocytes might have been different from those we were able to count after cell divisions, as a result of chemical activation. It has been previously reported that parthenogenetic [34,35,63] and SCNT bovine embryos [64,65] show a high incidence of abnormal karyotypes, after DMAP activation. Some authors have attributed these anomalies to the accelerated pronuclear formation and premature DNA synthesis observed in DMAP-activated oocytes, followed by karyokinesis without cytokinesis during the first cell cycle [35]. Such observations correlate with our results, which showed 55% of anomalies, while IVF controls only showed 15%. In the future, this new technique could be modified to allow the replication of blastomeres containing a unique chromosome, leading to a simple way of individual chromosome transfer which could have a wide range of applications.

**Table 3:** Ploidy of micronucleus injected bovine embryos

**Treatment**	**N**	**Spreads**	**Embryos**		
			**Less than 15 chromosomes (%)**	**Euploid (%)**	**Others (%)**
**Micronucleus- injected**	11	18	11 (100)^a^	0^a^	0^a^
**Parthenogenetic**	20	52	0^b^	9 (45)^b^	11 (55)^b^
**IVF**	20	49	0^b^	15 (75)^b^	3 (15)^a^

Micronucleus- injected (embryos generated from enucleated oocytes injected with one micronucleus and parthenogenetically activated), Parthenogenetic (parthenogenetically activated oocytes), IVF (embryos generated by *in vitro* fertilization). %: percentages over total embryos. Euploid: haploid + diploid. Others: tetraploid + mixoploid + aneuploid.

^a,b^:Values with different superscripts in the same column significantly differ (p≤0.05).

## Conclusions

In this study we have demonstrated that bovine ooplasts are capable of replicating micronuclei with a low number of chromosomes. The number of replicated chromosomes in each micronucleus was confirmed to be between 1 and 13. Moreover, it was observed that some of the transferred micronuclei expressed a foreign DNA, which means that it is not necessary for the oocyte to have an entire nucleus to allow the expression of a transgene. For the first time, MMCT was used to produce SCNT embryos, using micronuclei as nuclear donors. In conclusion, we have developed a new method to clone a small number of chromosomes, which could be a useful tool to transfer individual chromosomes and to target transgenesis to a specific area of the genome. In the field of domestic animals production, it could be a strategy for sex chromosomes interchange. It would make it possible to exchange an X chromosome for a Y chromosome, which could have been previously modified to carry a transgene of interest. In this way, the resulting male would be able to produce millions of gametes instead of hundreds and would have the transgene integrated into the Y chromosome, which has a few genes and therefore reduced position effects.

## Methods

Unless otherwise indicated, all chemicals were purchased from Sigma Chemical Company (St. Louis, MO, USA).

### Cumulus–oocyte complexes (COCs) collection and in vitro maturation (IVM)

Cow ovaries were transported from a local slaughterhouse to the laboratory in a thermo container at 24 to 27°C. Cumulus oocyte complexes (COCs) were aspirated from follicles with a diameter of 2–8 mm into Dulbecco’s phosphate-buffered saline (DPBS; 14287–072; Gibco BRL, Grand Island, NY, USA) containing 10% v/v fetal bovine serum (FBS; 10499–044; Gibco BRL) and 1% v/v antibiotic–antimycotic (ATB; 15240–096; Gibco BRL). Oocytes covered with at least three layers of granulosa cells were selected for IVM. The maturation medium was bicarbonate-buffered TCM-199 (31100–035; Gibco BRL), containing 2 mM glutamine (G-8540), 10% v/v FBS, 2 mg/ml follicle-stimulating hormone (NIH-FSH-P1; Folltropin; Bioniche, Belleville, Ontario, Canada), 0.3 mM sodium pyruvate (P2256), 100 mM cysteamine (M9768), and 1% v/v ATB. Groups of 25 COCs were *in vitro* matured in 100 μL droplets of maturation medium covered with mineral oil (M8410), at 39°C in a humidified atmosphere of 6% CO_2_ in air. After 21–24 h of IVM, cumulus cells were removed from COCs by vortexing for 3 min in 1 mg/ml hyaluronidase solution (H-4272) and washed three times in HEPES-TALP. Oocytes with an extruded first polar body (PB) were selected for enucleation and intracytoplasmic micronucleus injection or chemical activation.

### Preparation of donor cells

Somatic cell cultures were established from slaughtered cow ovaries. Ovarian explants were cultured in 35x100 mm culture dishes (353001; Falcon, USA) with Dulbecco modified Eagle medium (DMEM; 11885 Low glucose; Invitrogen, Grand Island, NY, USA) supplemented with 10% v/v FBS and 3% v/v ATB, at 39°C in a humidified atmosphere of 6% CO_2_ in air. After about 2 weeks, fibroblast monolayers reached confluence and were removed and placed into new culture dishes. For passages, cells were washed with DPBS and digested with 0.05% trypsin-EDTA (25300; Gibco, Grand Island, NY) for 5 min at 37°C. The reaction was terminated by adding supplemented DMEM and the collected cells were resuspended and divided into new dishes.

### Induction of somatic micronucleate cells

Ovarian bovine fibroblasts at approximately 80% confluence with less than 5 passages were blocked in metaphase by incubation with 0.05 or 0.1 μg/ml demecolcine (DMC; D1925); or 0.05 μg/ml colchicine (Col; C3915) in DMEM for 46 or 48 h. In order to complete mitotic arrest, some cells were then incubated with 2 mg/ml mitomycin (Mit; M-4287) for 2 h. The cells were subsequently washed with DPBS and, in all cases, were incubated in DMEM medium supplemented with 10 to 15 μg/ml cytochalasin B (CB; C6762) for 1 h. The six treatments assayed were: DMC 0.05 μg/ml; DMC 0.05 μg/ml + Mit; DMC 0.1 μg/ml; DMC 0.1 μg/ml + Mit; Col 0.05 μg/ml; and Col 0.05 μg/ml + Mit. All treated cells were trypsinized and immediately centrifuged at 1200 r.p.m for 5 min, at 37°C. The pellet was resuspended in 200 μl of FBS-free DMEM medium and the resulting suspension containing micronucleate cells was transferred to 100 μl droplets of TALP-Hepes with 1 mg/ml of Hoechst Bisbenzimide 33342 (H33342, B-2261). After 10 min, cells were observed under a fluorescence microscope to analyze the percentage of micronucleate cells and the number of micronuclei per cell. For injection procedures, cells were exposed to DMC 0.05 μg/ml + Mit + CB 10 μg/ml treatment, then trypsinized and immediately used as nuclear donors as described below.

### DNA construction

The plasmid used was pCX-EGFP kindly provided by Dr. Masaru Okabe (Osaka University, Osaka, Japan) that contains the enhanced green fluorescent protein gene (*egfp*) under the chimeric cytomegalovirus-IE-chicken β-actin enhancer-promoter control [66]. The plasmid was linearized by Hind III digestion.

### Enucleation and intracytoplasmic micronucleus injection

After 21 h of IVM, MII oocytes were denuded and stained with 1 mg/ml of H 33342 for 10 min. Immediately, oocytes were transferred into 100 μl droplets of Hepes-TALP supplemented with 0.3 g/ml BSA, under mineral oil, in 100x20 mm tissue culture dishes (430167; Corning, NY, USA) and mechanically enucleated using a Narishige hydraulic micromanipulator (Narishige Sci., Tokyo, Japan) mounted on a Nikon Eclipse E-300 microscope (Nikon, Melville, NY, USA). Enucleation was performed using a 20 μm internal diameter pipette. Metaphase chromosomes were visualized under ultraviolet light (<10 sec) and aspirated into the pipette with a minimal volume of ooplasm. Chromosome removal was confirmed by the presence of stained MII chromosomes inside the pipette. Donor somatic cells were transferred to TALP-Hepes droplets supplemented with 10 μg/ml CB and 1 mg/ml of H 33342. After 10 min, micronucleate cells were identified and transferred to 3 μl droplets of 10% v/v polyvinylpyrrolidone solution (PVP; 99219; Irvine Sci., Irvine, CA, USA) in HEPES-TALP, with or without 50 ng/μl pCX-EGFP for injection. After an incubation of at least 5 min, the cell membrane was broken by gentle aspiration in and out of the injection pipette (inner diameter of 7 μm) and a single micronucleus was injected into the cytoplasm of each enucleated oocyte (Micronucleus- injected groups). Aspiration was used to break the oolemma. The somatic micronucleus and the aspirated ooplasm were then expelled into the oocyte with a minimal volume of PVP (<10 pl). Enucleated and some parthenogenetic controls were injected with 50 ng/μl pCX-EGFP in 10% v/v PVP into the ooplasm, using a volume that was equivalent to that used for to the injection of micronuclei (<10 pl). A parthenogenetic control (non injected) was also included. After injection, all groups were subjected to chemical activation as described below.

### Chemical activation

Metaphase II oocytes and Micronucleus- injected embryos were treated with 5 μM ionomycin (I24222; Invitrogen, Carlsbad, CA, USA) in HEPES-TALP for 4 min, followed by incubation for 3 h in 1.9 mM 6-DMAP, (D2629) diluted in TCM-199 medium. Afterwards, oocytes were thoroughly washed in HEPES-TALP and cultured as described below. Prior to activation, Micronucleus- injected embryos were held for 2 h in 100 μl droplets of TCM-199 to allow the reprogramming events.

### IVF procedure

The IVF procedure was previously described by Brackett and Oliphant [67]. Briefly, frozen semen was thawed in a 37°C water bath for 30 sec. The sperm were washed twice by centrifugation at 490g x 5 min with Brackett’s defined medium. Sperm concentration was adjusted to 20x10^6^/ml and sperm were then coincubated for 5 h with COCs in Brackett’s fertilization medium. Afterward, presumptive zygotes were washed several times in HEPES-TALP and *in vitro* cultured as described below.

### *In vitro* culture

Embryos were *in vitro* cultured in 50 μl droplets of synthetic oviductal fluid (SOF) [68] modified by Holm et al. [69] containing 2.5% v/v FBS under mineral oil, at 39°C in a humidified atmosphere of 6% CO_2_ in air. Cleavage stage was evaluated at day 2 of *in vitro* development.

### Evaluation of pCX-EGFP expression in embryos

Embryos injected with pCX-EGFP were briefly exposed to blue light using an excitation- filter at 488 nm and an emission-filter at 530 nm to determine *egfp* expression at day 4 of *in vitro* development.

### Assessment of nuclear replication

Forty-eight hours after activation, Micronucleus- injected and Parthenogenetic embryos were transferred to Hepes-TALP droplets and treated with 1.5 mg/ml pronase for 3 min to remove all zonae pellucidae. Afterward, embryos were fixed with methanol: acetic acid solution (3:1, v: v) and DNA was stained with DAPI (D9542). Nuclei were visualized and counted using UV light under a fluorescence microscope. Embryos with more than one nucleus were considered to have replicated their DNA.

### Chromosomal analysis

Forty-eight hours after activation, Micronucleus- injected, Parthenogenetic, and IVF embryos were cultured for 6 h in SOF medium containing 1.25 μg/ml colchicine (C3915) and transferred to a trisodium citrate hypotonic solution (F71497; 0.9 % w/v in distilled water) for 13 min at 37°C. Subsequently, embryos were placed on a clean glass slide in a small volume of medium and a methanol: acetic acid solution (3:1, v: v) was applied. After air drying, fixed embryos were stained with 5% v/v Giemsa solution (1.09204.1002; Merck, Darmstadt, Germany) in distilled water for 10 min. Chromosome spreads were examined under a 100X oil magnification objective and chromosomal complements were determined for each embryo which was classified as: less than 15 chromosomes, euploid (1n and 2n), and others, which included tetraploid (4n), mixoploid (embryos with blastomeres of different ploidies), and aneuploid. Only those spreads which were clearly in metaphase were analyzed.

### Statistical analysis

Each experiment was repeated at least three times. Differences between treatments in each experiment were determined by Fisher’s exact test using Graph Pad PRISM® software 5.01 version. Differences between media of micronuclei per cell were analyzed for significance using the one way ANOVA. For all analyses a difference of p≤0.05 was considered to be significant.

### Experimental design

**Figure 3:**
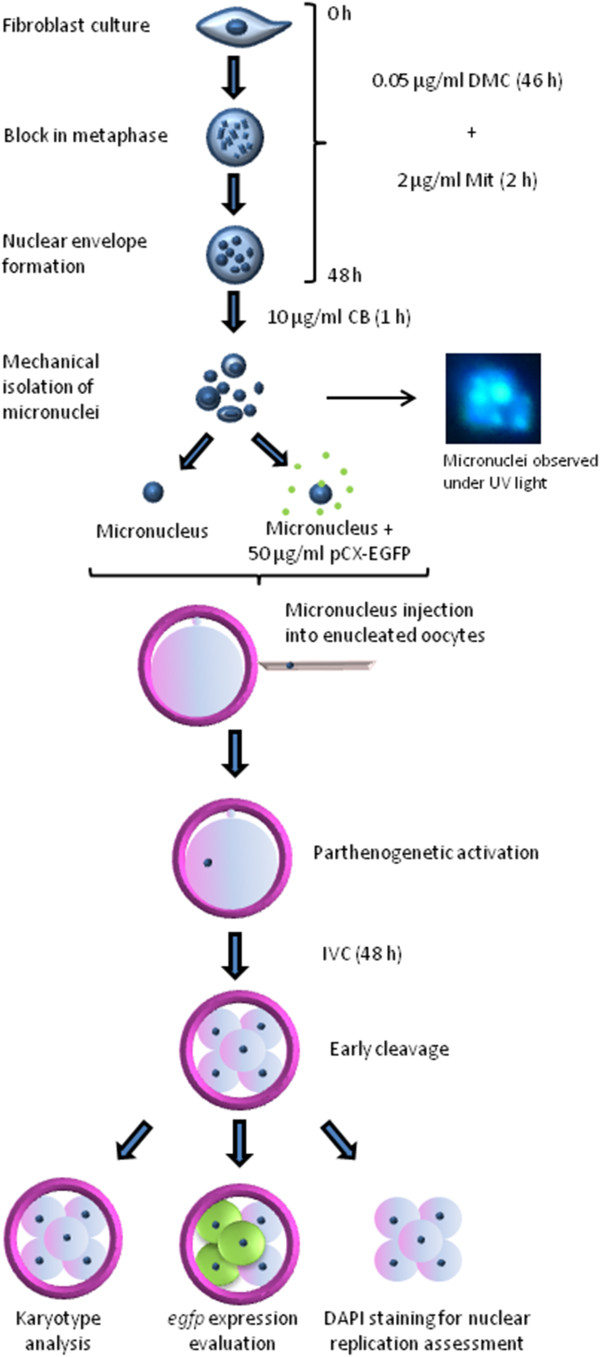
**Methodological design.** Micronuclei formation was induced by incubation in 0.05 μg/ml demecolcine for 46 h followed by 2 mg/ml mitomycin for 2 h. Cells were then treated with 10 μg/ml cytochalasin B for 1 h. MII oocytes were mechanically enucleated and injected with one somatic micronucleus, which were previously exposed or not to pCX-EGFP. After 2 h, oocytes and reconstituted embryos were chemically activated. After 48 h of IVC, cleavage stage was assessed. Moreover, the DNA from some embryos was stained with DAPI for evaluation of nuclear replication and others were karyotyped. At day 4 of IVC, *egfp* expression was evaluated.

In experiment 1, six different treatments were assayed on primary fibroblast cultures to induce micronucleus formation. In experiment 2, the best treatment from experiment 1 was employed to generate micronuclei, which were used as nuclear donors of reconstituted embryos. The methodology is shown in Figure 3. Briefly, micronucleus formation was induced by incubation in 0.05 μg/ml demecolcine for 46 h followed by 2 mg/ml mitomycin for 2 h. Cells were then treated with 10 μg/ml cytochalasin B for 1 h. Metaphase II oocytes were mechanically enucleated and injected with one somatic micronucleus, which had been previously exposed or not to pCX-EGFP. Enucleated oocytes and parthenogenetic controls were injected with 50 ng/μl pCX-EGFP. A non injected parthenogenetic control was also included. After 2 h, oocytes and reconstituted embryos were chemically activated. Cleavage stage and *egfp* expression were evaluated. Additionally, DNA replication from some Micronucleus- injected and Parthenogenetic embryos was confirmed by DAPI staining. In experiment 3, Micronucleus- injected, Parthenogenetic and *in vitro* fertilized (IVF) embryos were karyotyped. Methodological design is summarized in Figure 3.

## Competing interests

The authors declare that they have no competing interests.

## Authors’ contributions

DS and NC designed the experiments and NC, RB and MIH performed all the experiments. NC, RB and DS performed the data analysis. NC wrote the manuscript and DS is responsible for the scientific content of the manuscript. All authors have read and approved the final manuscript.

## References

[B1] Murray AW Szostak JW Construction of artificial chromosomes in yeast Nature 1983 305 189 193 10.1038/305189a0 6350893

[B2] Burke DT Carle GF Olson MV Cloning of large segments of exogenous DNA into yeast by means of artificial chromosome vectors Science 1987 236 806 812 10.1126/science.3033825 3033825

[B3] Harrington JJ Van BG Mays RW Gustashaw K Willard HF Formation of de novo centromeres and construction of first-generation human artificial microchromosomes Nature Genet 1997 15 345 355 10.1038/ng0497-345 9090378

[B4] Kuroiwa Y Tomizuka K Shinohara T Kazuki Y Yoshida H Ohguma A Yamamoto T Tanaka S Oshimura M Ishida I Manipulation of human minichromosomes to carry greater than megabase-sized chromosome inserts Nat Biotechnol 2000 18 1086 1090 10.1038/80287 11017048

[B5] Ege T Ringertz NR Preparation of microcells by enucleation of micronucleate cells Exp Cell Res 1974 87 378 382 10.1016/0014-4827(74)90494-7 4370277

[B6] Schor SL Johnson RT Mullinger AM Perturbation of mammalian cell division. II. Studies on the isolation and characterization of human mini segregant cells J Cell Sci 1975 19 281 303 53236 10.1242/jcs.19.2.281

[B7] Fournier RE Ruddle FH Microcell-mediated transfer of murine chromosomes into mouse, Chinese hamster, and human somatic cells Proc Natl Acad Sci USA 1977 74 319 23 10.1073/pnas.74.1.319 264685 PMC393251

[B8] Suzuki N Itou T Hasegawa Y Okazaki T Ikeno M Cell to cell transfer of the chromatin-packaged human beta-globin gene cluster Nucleic Acids Res 2010 38 e33 10.1093/nar/gkp1168 20007595 PMC2836578

[B9] Kazuki Y Hiratsuka M Takiguchi M Osaki M Kajitani N Hoshiya H Hiramatsu K Yoshino T Kazuki K Ishihara C Takehara S Higaki K Nakagawa M Takahashi K Yamanaka S Oshimura M Complete genetic correction of ips cells from Duchenne muscular dystrophy Mol Ther 2010 18 386 93 10.1038/mt.2009.274 19997091 PMC2839293

[B10] Saxon PJ Srivatsan ES Stanbridge EJ Introduction of human chromosome 11 via microcell transfer controls tumorigenic expression of HeLa cells EMBO J 1986 5 3461 6 2881780 10.1002/j.1460-2075.1986.tb04670.x PMC1167381

[B11] Casey G Plummer S Hoeltge G Scanlon D Fasching C Stanbridge EJ Functional evidence for a breast cancer growth suppressor gene on chromosome 17 Hum Mol Genet 1993 2 1921 7 10.1093/hmg/2.11.1921 8281156

[B12] Flanagan JM Healey S Young J Whitehall V Trott DA Newbold RF Chenevix-Trench G Mapping of a candidate colorectal cancer tumor-suppressor gene to a 900-kilobase region on the short arm of chromosome 8 Genes Chromosomes Cancer 2004 40 247 60 10.1002/gcc.20039 15139003

[B13] Cheung AK Lung HL Ko JM Cheng Y Stanbridge EJ Zabarovsky ER Nicholls JM Chua D Tsao SW Guan XY Lung ML Chromosome 14 transfer and functional studies identify a candidate tumor suppressor gene, mirror image polydactyly 1, in nasopharyngeal carcinoma Proc Natl Acad Sci USA 2009 106 14478 83 10.1073/pnas.0900198106 19667180 PMC2732794

[B14] Dafou D Grun B Sinclair J Lawrenson K Benjamin EC Hogdall E Kruger-Kjaer S Christensen L Sowter HM Al-Attar A Edmondson R Darby S Berchuck A Laird PW Pearce CL Ramus SJ Jacobs IJ Gayther SA Microcell-mediated chromosome transfer identifies EPB41L3 as a functional suppressor of epithelial ovarian cancers Neoplasia 2010 12 579 89 20651987 10.1593/neo.10340 PMC2907584

[B15] Notaridou M Quaye L Dafou D Jones C Song H Høgdall E Kjaer SK Christensen L Høgdall C Blaakaer J McGuire V Wu AH Van Den Berg DJ Pike MC Gentry-Maharaj A Wozniak E Sher T Jacobs IJ Tyrer J Schildkraut JM Moorman PG Iversen ES Jakubowska A Mędrek K Lubiński J Ness RB Moysich KB Lurie G Wilkens LR Carney ME Common alleles in candidate susceptibility genes associated with risk and development of epithelial ovarian cancer Int J Cancer 2011 128 2063 74 10.1002/ijc.25554 20635389 PMC3098608

[B16] Nakabayashi K Ogino H Michishita E Satoh N Ayusawa D Introduction of chromosome 7 suppresses telomerase with shortening of telomeres in a human mesothelial cell line Exp Cell Res 1999 252 376 82 10.1006/excr.1999.4619 10527627

[B17] Backsch C Wagenbach N Nonn M Leistritz S Stanbridge E Schneider A Dürst M Microcell-mediated transfer of chromosome 4 into HeLa cells suppresses telomerase activity Genes Chromosomes Cancer 2001 31 196 8 10.1002/gcc.1134 11319808

[B18] Abe S Tanaka H Notsu T Horike S Fujisaki C Qi DL Ohhira T Gilley D Oshimura M Kugoh H Localization of an hTERT repressor region on human chromosome 3p21.3 using chromosome engineering Genome Integr 2010 26 6 20678252 10.1186/2041-9414-1-6 PMC2907559

[B19] Qi DL Ohhira T Fujisaki C Inoue T Ohta T Osaki M Ohshiro E Seko T Aoki S Oshimura M Kugoh H Identification of PITX1 as a TERT suppressor gene located on human chromosome 5 Mol Cell Biol 2011 31 1624 36 10.1128/MCB.00470-10 21300782 PMC3126332

[B20] Klein CB Conway K Wang XW Bhamra RK Lin XH Cohen MD Annab L Barrett JC Costa M Senescence of nickel-transformed cells by an X chromosome: possible epigenetic control Science 1991 251 796 9 10.1126/science.1990442 1990442

[B21] Kugoh H Fujiwara M Kihara K Fukui I Horikawa I Schulz TC Oshimura M Cellular senescence of a human bladder carcinoma cell line (JTC-32) induced by a normal chromosome 11 Cancer Genet Cytogenet 2000 116 158 63 10.1016/S0165-4608(99)00138-7 10640149

[B22] Poignée M Backsch C Beer K Jansen L Wagenbach N Stanbridge EJ Kirchmayr R Schneider A Dürst M Evidence for a putative senescence gene locus within the chromosomal region 10p14-p15 Cancer Res 2001 61 7118 21 11585743

[B23] Jeggo PA Hafezparast M Thompson AF Broughton BC Kaur GP Zdzienicka MZ Athwal RS Localization of a DNA repair gene (XRCC5) involved in double-strand-break rejoining to human chromosome 2 Proc Natl Acad Sci USA 1992 89 6423 7 10.1073/pnas.89.14.6423 1631138 PMC49513

[B24] Chen DJ Marrone BL Nguyen T Stackhouse M Zhao Y Siciliano MJ Regional assignment of a human DNA repair gene (XRCC5) to 2q35 by X-ray hybrid mapping Genomics 1994 21 423 7 10.1006/geno.1994.1287 8088837

[B25] Whitney M Thayer M Reifsteck C Olson S Smith L Jakobs PM Leach R Naylor S Joenje H Grompe M Microcell mediated chromosome transfer maps the Fanconi anaemia group D gene to chromosome 3p Nat Genet 1995 11 341 3 10.1038/ng1195-341 7581463

[B26] Upender MB Habermann JK McShane LM Korn EL Barrett JC Difilippantonio MJ Ried T Chromosome transfer induced aneuploidy results in complex dysregulation of the cellular transcriptome in immortalized and cancer cells Cancer Res 2004 64 6941 9 10.1158/0008-5472.CAN-04-0474 15466185 PMC4772432

[B27] Nawata H Kashino G Tano K Daino K Shimada Y Kugoh H Oshimura M Watanabe M Dysregulation of gene expression in the artificial human trisomy cells of chromosome 8 associated with transformed cell phenotypes PLoS One 2011 6 e25319 10.1371/journal.pone.0025319 21980425 PMC3183047

[B28] Meguro M Mitsuya K Sui H Shigenami K Kugoh H Nakao M Oshimura M Evidence for uniparental, paternal expression of the human GABAA receptor subunit genes, using microcell-mediated chromosome transfer Hum Mol Genet 1997 6 2127 33 10.1093/hmg/6.12.2127 9328477

[B29] Kugoh H Mitsuya K Meguro M Shigenami K Schulz TC Oshimura M Mouse A9 cells containing single human chromosomes for analysis of genomic imprinting DNA Res 1999 6 165 72 10.1093/dnares/6.3.165 10470847

[B30] Inoue J Mitsuya K Maegawa S Kugoh H Kadota M Okamura D Shinohara T Nishihara S Takehara S Yamauchi K Schulz TC Oshimura M Construction of 700 human/mouse A9 monochromosomal hybrids and analysis of imprinted genes on human chromosome 6 J Hum Genet 2001 46 137 45 10.1007/s100380170101 11310581

[B31] Doherty AM Fisher EM Microcell-mediated chromosome transfer (MMCT): small cells with huge potential Mamm Genome 2003 14 583 92 10.1007/s00335-003-4002-0 14629108

[B32] Anderson MJ Stanbridge EJ Tumor suppressor genes studied by cell hybridization and chromosome transfer FASEB J 1993 7 826 833 8344482 10.1096/fasebj.7.10.8344482

[B33] Meaburn KJ Parris CN Bridger JM The manipulation of chromosomes by mankind: the uses of microcell-mediated chromosome transfer Chromosoma 2005 114 263 74 10.1007/s00412-005-0014-8 16133353

[B34] Winger QA De La Fuente R King WA Armstrong DT Watson AJ Bovine parthenogenesis is characterized by abnormal chromosomal complements: implications for maternal and paternal co-dependence during early bovine development Dev Genet 1997 21 160 6 10.1002/(SICI)1520-6408(1997)21:2<160::AID-DVG5>3.0.CO;2-5 9332973

[B35] De La Fuente R King WA Developmental consequences of karyokinesis without cytokinesis during the first mitotic cell cycle of bovine parthenotes Biol Reprod 1998 58 952 62 10.1095/biolreprod58.4.952 9546725

[B36] Hao YH Lai LX Liu ZH Im GS Wax D Samuel M Murphy CN Sutovsky P Prather RS Developmental competence of porcine parthenogenetic embryos relative to embryonic chromosomal abnormalities Mol Reprod Dev 2006 73 77 82 10.1002/mrd.20358 16224773

[B37] Angell RR Templeton AA Aitken RJ Chromosome studies in human in vitro fertilization Hum Genet 1986 72 333 9 10.1007/BF00290960 3699823

[B38] Munné S Lee A Rosenwaks Z Grifo J Cohen J Diagnosis of major chromosome aneuploidies in human preimplantation embryos Hum Reprod 1993 8 2185 91 8150922 10.1093/oxfordjournals.humrep.a138001

[B39] Pellestor F Girardet A Andréo B Arnal F Humeau C Relationship between morphology and chromosomal constitution in human preimplantation embryo Mol Reprod Dev 1994 39 141 6 10.1002/mrd.1080390204 7826614

[B40] Clouston HJ Herbert M Fenwick J Murdoch AP Wolstenholme J Cytogenetic analysis of human blastocysts Prenat Diagn 2002 22 1143 52 10.1002/pd.502 12454974

[B41] Shaver EL Carr DH Chromosome abnormalities in rabbit blastocysts following delayed fertilization J Reprod Fertil 1967 14 415 20 10.1530/jrf.0.0140415 6066497

[B42] Modlinski JA Transfer of embryonic nuclei to fertilized mouse eggs and development of tetraploid blastocysts Nature 1978 273 466 7 10.1038/273466a0 566383

[B43] Vichera G Olivera R Sipowicz P Radrizzani M Salamone D Sperm genome cloning used in biparental bovine embryo reconstruction Reprod Fertil Dev 2011 23 769 79 21791178 10.1071/RD10252

[B44] Vichera G Olivera R Salamone D Oocyte genome cloning used in biparental bovine embryo reconstruction Zygote 2012 5 1 9 22475091 10.1017/S0967199412000081

[B45] McGrath J Solter D Nuclear transplantation in mouse embryo by microsurgery and cell fusion Science 1983 220 1300 1302 10.1126/science.6857250 6857250

[B46] Wilmut I Schnieke AE McWhir J Kind AJ Campbell KH Viable offspring derived from fetal and adult mammalian cells Nature 1997 385 810 813 10.1038/385810a0 9039911

[B47] Dominko T Mitalipova M Haley B Beyhan Z Memili E McKusick B First NL Bovine oocyte cytoplasm supports development of embryos produced by nuclear transfer of somatic cell nuclei from various mammalian species Biol Reprod 1999 60 1496 1502 10.1095/biolreprod60.6.1496 10330111

[B48] Wakayama T Yanagimachi R The first polar body can be used for the production of normal offspring in mice Biol Reprod 1998 59 100 4 10.1095/biolreprod59.1.100 9674999

[B49] Meng Q Bai C Liu Y Wu X Bunch TD Li GP In vitro development and chromosomal configuration of bovine somatic cloned embryos with nonenucleated metaphase II oocytes Cell Reprogram 2010 12 481 90 10.1089/cell.2009.0114 20698786

[B50] Pereyra-Bonnet F Bevacqua R La Rosa I Sipowicz P Radrizzani M Fernandez-Martin R Salamone D Novel methods to induce exogenous gene expression in SCNT, parthenogenic and IVF preimplantation bovine embryos Transgenic Res 2011 20 1379 88 10.1007/s11248-011-9503-0 21431868

[B51] Katoh M Kazuki Y Kazuki K Kajitani N Takiguchi M Nakayama Y Nakamura T Oshimura M Exploitation of the interaction of measles virus fusogenic envelope proteins with the surface receptor CD46 on human cells for microcell-mediated chromosome transfer BMC Biotechnol 2010 10 37 10.1186/1472-6750-10-37 20444293 PMC2874513

[B52] Littlefield JW The use of drug-resistant markers to study the hybridization of mouse fibroblasts Exp Cell Res 1966 41 190 196 10.1016/0014-4827(66)90558-1 4952025

[B53] Horibata K Iwamoto Y Kuraoka I Jaspers NG Kurimasa A Oshimura M Ichihashi M Tanaka K Complete absence of Cockayne syndrome group B gene product gives rise to UV-sensitive syndrome but not Cockayne syndrome Proc Natl Acad Sci USA 2004 101 15410 5 10.1073/pnas.0404587101 15486090 PMC524447

[B54] De Lonlay P Mugnier C Sanlaville D Chantrel-Groussard K Bénit P Lebon S Chrétien D Kadhom N Saker S Gyapay G Romana S Weissenbach J Munnich A Rustin P Rötig A Cell complementation using Genebridge 4 human: rodent hybrids for physical mapping of novel mitochondrial respiratory chain deficiency genes Hum Mol Genet 2002 11 3273 81 10.1093/hmg/11.26.3273 12471054

[B55] Bevacqua RJ Pereyra-Bonnet F Olivera R Hiriart MI Sipowicz P Fernandez-Martín R Radrizzani M Salamone DF Production of IVF transgene-expressing bovine embryos using a novel strategy based on cell cycle inhibitors Theriogenology 2012 78 57 68 10.1016/j.theriogenology.2012.01.020 22494679

[B56] Boyle S Gilchrist S Bridger J Mahy N Ellis J Bickmore W The spatial organization of human chromosomes within the nuclei of normal and emerin-mutant cells Hum Mol Genet 2001 10 211 219 10.1093/hmg/10.3.211 11159939

[B57] Spann TP Goldman AE Wang C Huang S Goldman RD Alteration of nuclear lamin organization inhibits RNA polymerase II-dependent transcription J Cell Biol 2002 156 603 8 10.1083/jcb.200112047 11854306 PMC2174089

[B58] Hyttel P Viuff D Avery B Laurincik J Greve T Transcription and cell cycle-dependent development of intranuclear bodies and granules in two-cell bovine embryos J Reprod Fertil 1996 108 263 270 10.1530/jrf.0.1080263 9038785

[B59] Viuff D Hyttel P Avery B Vajta G Greve T Callesen H Thomsen PD Ribosomal ribonucleic acid is transcribed at the 4-cell stage in in vitro-produced bovine embryos Biol Reprod 1998 59 626 631 10.1095/biolreprod59.3.626 9716563

[B60] Memili E First NL Control of gene expression at the onset of bovine embryonic development Biol Reprod 1999 61 1198 1207 10.1095/biolreprod61.5.1198 10529265

[B61] Frei RE Schultz GA Church RB Qualitative and quantitative changes in protein synthesis occur at the 8–16-cell stage of embryogenesis in the cow J Reprod Fertil 1989 86 637 641 10.1530/jrf.0.0860637 2760892

[B62] Memili E Dominko T First NL Onset of transcription in bovine oocytes and preimplantation embryos Mol Reprod Dev 1998 51 36 41 10.1002/(SICI)1098-2795(199809)51:1<36::AID-MRD4>3.0.CO;2-X 9712315

[B63] Canel N Bevacqua R Fernández-Martín R Salamone DF Activation with ionomycin followed by dehydroleucodine and cytochalasin B for the production of parthenogenetic and cloned bovine embryos Cell Reprogram 2010 12 491 9 10.1089/cell.2009.0109 20698787

[B64] Bhak JS Lee SL Ock SA Mohana Kumar B Choe SY Rho GJ Developmental rate and ploidy of embryos produced by nuclear transfer with different activation treatments in cattle Anim Reprod Sci 2006 92 37 49 10.1016/j.anireprosci.2005.04.016 15979829

[B65] Yoo JG Choe SY Rho GJ Efficient production of cloned bovine embryos using cdc2 kinase inhibitor Reprod Domest Anim 2003 38 444 450 10.1046/j.0936-6768.2003.00461.x 14629666

[B66] Ikawa M Kominami K Yoshimura Y Tanaka K Nishimune Y Okabe M A rapid and non-invasive selection of transgenic embryos before implantation using green fluorescent protein (GFP) FEBS Lett 2005 375 125 128 7498460 10.1016/0014-5793(95)01162-8

[B67] Brackett B Oliphant G Capacitation of rabbit spermatozoa in vitro Biol Reprod 1975 12 260 274 10.1095/biolreprod12.2.260 1122333

[B68] Tervit H Whittingham D Rowson L Successful culture in vitro of sheep and cattle ova J Reprod Fertil 1972 30 493 497 10.1530/jrf.0.0300493 4672493

[B69] Holm P Booth PJ Schmidt MH Greve T Callesen H High bovine blastocyst development in a static in vitro production system using SOFaa medium supplemented with sodium citrate and myo-inositol with or without serum-proteins Theriogenology 1999 52 683 700 10.1016/S0093-691X(99)00162-4 10734366

